# Deciphering the role of FUS::DDIT3 expression and tumor microenvironment in myxoid liposarcoma development

**DOI:** 10.1186/s12967-024-05211-w

**Published:** 2024-04-26

**Authors:** Parmida Ranji, Emma Jonasson, Lisa Andersson, Stefan Filges, Manuel Luna Santamaría, Christoffer Vannas, Soheila Dolatabadi, Anna Gustafsson, Ola Myklebost, Joakim Håkansson, Henrik Fagman, Göran Landberg, Pierre Åman, Anders Ståhlberg

**Affiliations:** 1 Sahlgrenska Center for Cancer Research, Department of Laboratory Medicine, Institute of Biomedicine, Sahlgrenska Academy at University of Gothenburg, Gothenburg, Sweden; 2 Present Address: Wallenberg Centre for Molecular and Translational Medicine, University of Gothenburg, Gothenburg, Sweden; 3 Department of Oncology, Region Västra Götaland, Sahlgrenska University Hospital, Gothenburg, Sweden; 4 Department of Tumor Biology, Oslo University Hospital, Oslo, Norway; 5 Institute for Clinical Science, University of Bergen, Bergen, Norway; 6 RISE Unit of Biological Function, Division Materials and Production, RISE Research Institutes of Sweden, Borås, Sweden; 7 Department of Laboratory Medicine, Institute of Biomedicine, Sahlgrenska Academy at University of Gothenburg, Gothenburg, Sweden; 8 Department of Chemistry and Molecular Biology, Faculty of Science at University of Gothenburg, Gothenburg, Sweden; 9 Department of Clinical Pathology, Region Västra Götaland, Sahlgrenska University Hospital, Gothenburg, Sweden; 10 Department of Clinical Genetics and Genomics, Region Västra Götaland, Sahlgrenska University Hospital, Gothenburg, Sweden

**Keywords:** Extracellular matrix, FET fusion oncogenes, FUS::DDIT3, Microenvironment, Myxoid liposarcoma, Scaffold

## Abstract

**Background:**

Myxoid liposarcoma (MLS) displays a distinctive tumor microenvironment and is characterized by the *FUS::DDIT3* fusion oncogene, however, the precise functional contributions of these two elements remain enigmatic in tumor development.

**Methods:**

To study the cell-free microenvironment in MLS, we developed an experimental model system based on decellularized patient-derived xenograft tumors. We characterized the cell-free scaffold using mass spectrometry. Subsequently, scaffolds were repopulated using sarcoma cells with or without FUS::DDIT3 expression that were analyzed with histology and RNA sequencing.

**Results:**

Characterization of cell-free MLS scaffolds revealed intact structure and a large variation of protein types remaining after decellularization. We demonstrated an optimal culture time of 3 weeks and showed that FUS::DDIT3 expression decreased cell proliferation and scaffold invasiveness. The cell-free MLS microenvironment and FUS::DDIT3 expression both induced biological processes related to cell-to-cell and cell-to-extracellular matrix interactions, as well as chromatin remodeling, immune response, and metabolism. Data indicated that FUS::DDIT3 expression more than the microenvironment determined the pre-adipocytic phenotype that is typical for MLS.

**Conclusions:**

Our experimental approach opens new means to study the tumor microenvironment in detail and our findings suggest that FUS::DDIT3-expressing tumor cells can create their own extracellular niche.

**Supplementary Information:**

The online version contains supplementary material available at 10.1186/s12967-024-05211-w.

## Background

Myxoid liposarcoma (MLS) represents 20–30% of all liposarcomas and generally occur in deep soft tissues, most frequently in musculature of the extremities [1]. Genetically, MLS belongs to a group of more than ten different sarcoma entities all defined by FET (*FUS*, *EWSR1* and *TAF15*) fusion oncogenes, which are formed by the N-terminal part of FET genes fused to one of various transcription factor partners [2, 3]. Whereas the FET genes often can replace each other, the transcription factor partner is most often specific for each tumor entity [3]. MLS, specifically, is characterized by either the *FUS::DDIT3* or the less common *EWSR1::DDIT3* fusion oncogene [1]. The fusion oncogene is believed to be causative since MLS contains few additional mutations [4, 5]. FUS::DDIT3 expression generates MLS-like tumors in mice, using both transgenic models [6–8] or xenografting of human FUS::DDIT3-expressing cells [9–12]. These studies indicate that a mesenchymal progenitor cell is likely the cell of origin for tumor development and that FUS::DDIT3 mediates the MLS phenotype, but no attempts have succeeded in transforming non-malignant human cells into an MLS cell with *FUS::DDIT3* as the only driver mutation. Hence, the exact molecular mechanism of *FUS::DDIT3* in tumor cell transformation remains unclear.

The microenvironment plays an important role in tumor development, influencing all stages from initiation to invasion and metastasis [13, 14]. The microenvironment contains non-neoplastic cells, such as fibroblasts, immune cells and vascular cell types, as well as various extracellular molecules, including structural molecules, building up the extracellular matrix (ECM) [15]. Myxoid liposarcoma displays a distinct histology consisting of either small round or oval-shaped tumor cells that are surrounded by an ample myxoid matrix with thin-walled, branching blood vessels. The main components of myxoid ECMs are collagens but also glycosaminoglycans, such as hyaluronic acid and fibronectin, are present [16]. Myxoid liposarcomas also often contain lipoblasts [1], however, FUS::DDIT3 blocks terminal adipocytic differentiation [7, 11, 17]. High-grade MLS, associated with poor prognosis, is defined by hypercellularity and diminished myxoid matrix content [1]. The clinical relevance of microenvironmental features, including angiogenesis [18, 19], immune response [20, 21] and specific ECM components [22, 23] have been studied to some extent. However, the role of the microenvironment in MLS tumor development still remains unknown.

We hypothesized that both the FUS::DDIT3 expression and the microenvironment are important in MLS development. To study the cell-free microenvironment and its effect on tumor cells, we developed an experimental model system based on MLS scaffolds generated from decellularized patient-derived xenograft (PDX) tumors, which were repopulated with sarcoma cell lines. The protein composition of cell-free tumor scaffolds was characterized by mass spectrometry. To identify microenvironmentally induced gene expression profiles, we performed RNA sequencing on cells cultured in MLS scaffolds compared with cells cultured in traditional monolayers. To study the effect of FUS::DDIT3 expression, we compared gene expression signatures between scaffold-cultured cells with and without FUS::DDIT3 expression to determine the specific role of the fusion oncogene. Finally, we performed single-cell RNA sequencing on cells with and without FUS::DDIT3 cultured either in MLS scaffolds or as cell-derived xenografts, to characterize the gradual transition between tumor cell phenotypes. The applied approach allowed us to simultaneously assess the effects of FUS::DDIT3 expression and the cell-free MLS microenvironment.

## Materials and methods

### Xenograft models and myxoid liposarcoma scaffold generation

All in vivo experiments were performed in accordance with EU directive 2010/63. An MLS PDX model was maintained by transplanting tumor pieces of approximately 2 × 2 × 2 mm bilaterally into the flanks of 4 to 6 weeks old female BALB/c nude mice (Taconic, Borup, Denmark). Mice were sacrificed by isoflurane anesthesia, followed by incision of the right ventricle. For scaffold generation, tumors were surgically removed after euthanasia, frozen on dry ice and stored in − 80 °C until subsequent analysis. For cell-derived xenografts, 2 to 4 million cells were injected unilaterally, subcutaneously into the flanks of BALB/c nude mice. After 10–23 days, tumors were harvested.

For scaffold generation, collected PDX tumors were cut into pieces (6 × 6 × 6 mm) and washed twice for 6 h in decellularization buffer, consisting of distilled water containing 3.5 mM sodium dodecyl sulfate (Sigma-Aldrich, St. Louis, MO, USA), 3.1 mM sodium azide (VWR, Radnor, PA, USA), 5 mM 2H_2_O-Na_2_-EDTA (Sigma-Aldrich) and 0.4 mM phenylmethylsulfonyl fluoride (Sigma-Aldrich). Thereafter, the tumors were rinsed in the same buffer without sodium dodecyl sulfate for 15 min. Next, the scaffolds were washed for 72 h in distilled water exchanged every 12 h to remove remaining cell debris, followed by a 24 h wash in phosphate*-*buffered saline (PBS) solution (Medicago, Uppsala, Sweden) exchanged three times. All these wash steps were performed in a shaking incubator (Incu-Shaker 10L, Benchmark Scientific, Sayreville, NJ, USA) at 37 °C and 175 rpm. Sterilization of the scaffolds were performed by incubation in 0.1% peracetic acid (Sigma-Aldrich) in distilled water for 1 h at room temperature followed by a final wash in PBS containing 1% Antibiotic–Antimycotic (Thermo Fisher Scientific, Waltham, MA, USA) for 24 h in a shaking incubator at 37 °C and 175 rpm. Decellularized scaffold pieces were stored in PBS containing 3.1 mM sodium azide and 5 mM 2H_2_O-Na_2_-EDTA in 4 °C until later use. After complete decellularization, scaffolds were cut into smaller pieces (2 × 2 × 2 mm). Scaffold pieces were then soaked in cell culture media for about 30 min to remove residual storage buffer before repopulation.

To test for residual DNA in cell-free scaffolds, DNA was extracted using DNeasy Blood and Tissue purification kit (Qiagen, Hilden, Germany), according to the manufacturer’s instructions. DNA concentration was quantified by Qubit 3.0 fluorometer using the Qubit dsDNA HS Assay kit (both Thermo Fisher Scientific), according to the manufacturer’s instructions.

### Histological staining

Scaffolds were fixed in 4% phosphate-buffered formaldehyde, embedded in paraffin (both Histolab Products, Gothenburg, Sweden) and sectioned to 4.5 μm thickness by Microm Cool-Cut (Thermo Fisher Scientific). Tissue sections were deparaffinized with xylene (Histolab Products) and ethanol and rehydrated in water. Deparaffinized sections were stained with Mayer’s hematoxylin and eosin (Histolab Products), followed by dehydration with increasing concentrations of ethanol and mounting onto glass slides with Pertex (Histolab Products). Picro-Sirius Red (Abcam, Cambridge, United Kingdom) staining was performed according to the manufacturer’s instructions. Stained sections were scanned by Leica SCN400 scanner (Leica Microsystems, Wetzlar, Germany).

### Cell culture and scaffold repopulation

The fibrosarcoma cell lines HT1080 wild-type (WT, available at ATCC, Manassas, VA, USA) [24], HT1080 eGFP [25] and HT1080 FUS::DDIT3-eGFP [10] as well as MLS cell lines 2645‐94 and 1765‐92 [5, 26] were cultured in RPMI 1640 GlutaMAX medium supplemented with 5% fetal bovine serum, 100 U/mL penicillin and 100 μg/mL streptomycin (all Thermo Fisher Scientific), at 37 °C in 5% CO_2_. The HT1080 cells with either eGFP or FUS::DDIT3-eGFP were cultured in the presence of 500 µg/mL Geneticin (Thermo Fisher Scientific). Cell lines were regularly verified by cell line authentication tests (Eurofins Genomics, Ebersberg, Germany).

For repopulation of scaffolds, 3 × 10^5^ cells were seeded on top of each scaffold in a 48-well culture plate, containing 0.5 mL complete media. Visual inspection showed that lower cell seeding density repopulated the scaffold less efficiently, while higher cell seeding density resulted in massive cell growth on the plastic dish. Scaffolds were transferred into a new well 2 to 3 days after cell seeding. Each culture was inspected every fourth day and if cells started to expand into the plastic dish, the scaffold was transferred to a new well. Repopulated scaffolds were cultured for 1, 3 or 7 weeks before downstream analysis.

### Mass spectrometry

Cell-free scaffolds were homogenized using a scalpel in 200 µl storage buffer, containing 3.1 mM sodium azide, 0.5 mM EDTA (VWR) and PBS solution (Medicago) and thereafter forwarded for liquid chromatography-mass spectrometry/mass spectrometry analysis at the Proteomics Core Facility of Sahlgrenska Academy, University of Gothenburg (Gothenburg, Sweden).

Proteins were required to be expressed in all four MLS scaffold replicates with a coefficient of variation < 0.5 for further analysis. Functional protein classification was performed using the PANTHER web tool v16.0 [27, 28]. PANTHER overrepresentation test was used with Fisher’s exact test and false discovery rate for *p*-value correction. As background, all genes in the genome annotated as protein-coding by ENSEMBL was used.

### RNA sequencing

Total RNA was extracted using RNeasy Micro Kit or miRNeasy micro kit (both Qiagen) according to manufacturer’s instructions. RNA sequencing was performed according to the Smart-Seq2 protocol [29] with some modifications [30]. After sequencing, read alignment was performed using STAR RNA-seq aligner v2.6 [31] with ENSEMBL GRCh38 assembly as the reference genome. Read count matrices were generated using the HTSeq python framework v0.9.1 [32]. Differential expression was analyzed using the R package DESeq2 [33]. For additional details, see Additional file 1.

### Single-cell RNA sequencing

For MLS scaffolds, single cells were detached from the scaffold using 0.25% trypsin (Thermo Fisher Scientific). For xenografts, tumors were dissociated using collagenase/hyaluronidase (STEMCELL Technologies, Vancouver, Canada). Viable cells were enriched with Dead Cell Removal Kit (Miltenyi Biotec, Bergisch Gladbach, Germany), according to the manufacturer’s instructions.

Singe cell suspensions were processed immediately and incorporated into Single Cell 3’ gel beads on a Chromium instrument (10 × Genomics, Pleasanton, CA, USA). Single-cell data analysis was performed using Cell Ranger v4.1.1 (10 × Genomics) and the resulting barcode matrices were analyzed in R using the Seurat package v4.0.3 [34]. For additional details, see Additional file 1.

### Statistical analysis

Statistical analysis of cellular growth in scaffolds was performed using Prism (GraphPad Software, San Diego, CA, USA). Unpaired Student’s t-test was used for two groups whereas one-way ANOVA with Tukey’s multiple comparison test was used for three groups. Significant results were considered for *p* ≤ 0.05.

## Results

### Development of an experimental model system based on the myxoid liposarcoma cell-free microenvironment that support cell growth and infiltration

**Fig. 1:**
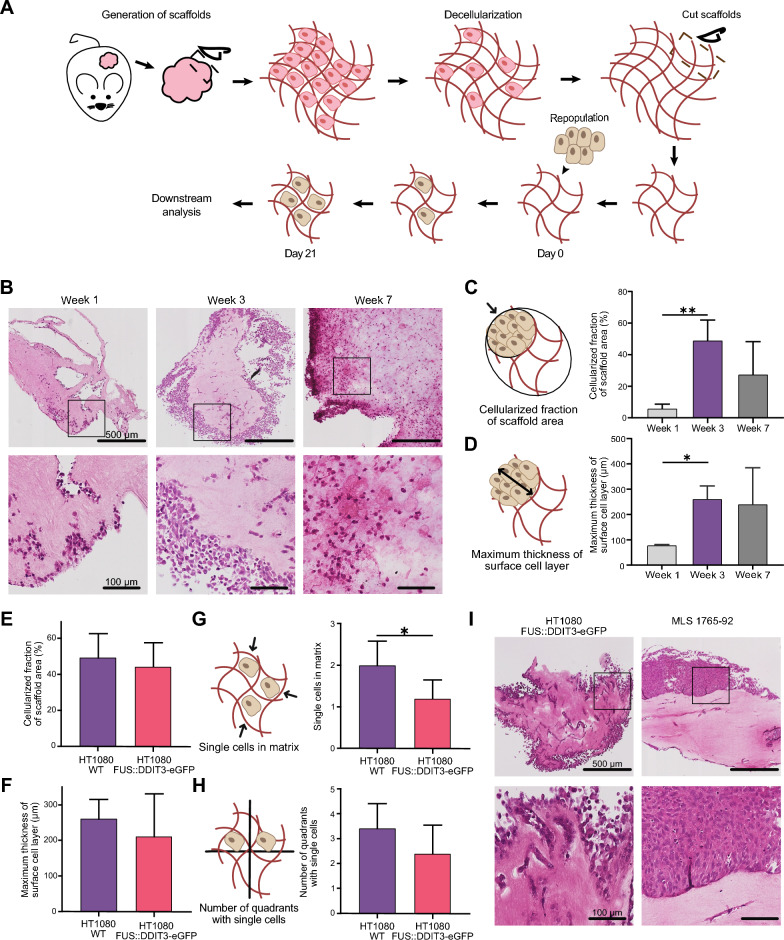
Myxoid liposarcoma scaffolds as an in vivo-like growth model system to study the effect of fusion oncogene *FUS::DDIT3*. **A** The tumor tissue was cut into pieces (~ 6 × 6 × 6 mm) and then decellularized using two rounds of detergent washing and cut into smaller pieces (~ 2 × 2 × 2 mm). Cell-free scaffolds were repopulated by adding sarcoma cells of interest followed by 3 weeks of growth before downstream analysis. **B** Hematoxylin and eosin staining of myxoid liposarcoma (MLS) scaffolds repopulated with HT1080 wild-type (WT) cells cultured for 1, 3 and 7 weeks. Images below are representative magnifications. **C**, **D** Comparison of HT1080 WT cells after 1, 3 and 7 weeks of culture in scaffolds and quantification of **C** cellularized fraction of the scaffold area, calculated as the area covered by cells divided by the total area, and **D** maximum thickness of surface cell layer. Mean ± SEM is shown, n = 3–7. *p ≤ 0.05, **p ≤ 0.01, one-way ANOVA with Tukey’s multiple comparison test. **E**–**H** Comparison of HT1080 cells with and without ectopic FUS::DDIT3-eGFP expression cultured in scaffolds for 3 weeks and quantification of **E** cellularized fraction of the scaffold area, **F** maximum thickness of surface cell layer, **G** the number of single cells migrating into the matrix, where the single cells inside the scaffold area was calculated (0 = 0 cells, 1 = 1–20 cells, 2 = 20–50 cells, 3 ≥ 51 cells), and **H** number of quadrants with single cells, where the scaffold area was divided into four quadrants and the number of quadrants containing at least 5 single cells were calculated. Mean ± SEM is shown, n = 5–7. *p < 0.05, Student’s t-test. **I** Hematoxylin and eosin staining of scaffolds repopulated with HT1080 cells with ectopic FUS::DDIT3-eGFP expression and MLS cell line 1765-92 cells both cultured for 3 weeks. Images below are representative magnifications

To enable detailed studies of the MLS microenvironment, we developed a tumor-derived three-dimensional experimental model system (Fig. 1A). Myxoid liposarcoma tissue from PDXs was cut into pieces (~ 6 × 6 × 6 mm) and decellularized by repeated washes with mild detergents to generate MLS scaffolds, i.e., cell-free MLS microenvironments. Two rounds of washing were sufficient to remove all cell debris as shown by hematoxylin and eosin staining (Additional file 2: Fig. S1A). In addition, we quantified the DNA concentration to be less than 2 nanogram per milligram tissue, which was considered to indicate a cell-free tissue [35]. Next, we showed that the scaffold structure was intact after the decellularization process using Picro-Sirius red staining for collagen (Additional file 2: Fig. S1B). Before repopulation, scaffolds were cut into smaller pieces (~ 2 × 2 × 2 mm), enabling up to 100 individual scaffolds to be generated from each PDX tumor for downstream experiments.

To determine the ability of cell-free MLS scaffolds to support cell growth and infiltration, we repopulated them with wild-type (WT) cells of the HT1080 fibrosarcoma cell line. We determined the optimal cultivation time by allowing HT1080 WT cells to grow in the scaffolds for 1, 3 and 7 weeks followed by hematoxylin and eosin staining (Fig. 1B). After 1 week, cells mainly grew on the scaffold surface, while cells also infiltrated the scaffolds after 3 weeks. After 7 weeks of growth, some cells had condensed cytoplasm and nuclei, and occasionally cells became necrotic (Fig. 1B). We quantified the cellularized fraction of the scaffold area and the maximum thickness of the surface cell layer (Fig. 1C, D) and found that maximum cellularity and infiltration was obtained at 3 weeks with no additional increase at 7 weeks. Instead, we observed larger variability between individual scaffolds after 7 weeks of growth. Hence, we used 3 weeks to repopulate MLS scaffolds for subsequent experiments.

### FUS::DDIT3 expression decreases cell infiltration in myxoid liposarcoma scaffolds

To determine the effects of FUS::DDIT3 expression on cell growth and infiltration, we compared the growth of HT1080 cells with and without ectopic FUS::DDIT3-eGFP expression. In addition to the cellularized fraction of the scaffold area and maximum thickness of the surface cell layer (Fig. 1E, F), we also quantified the number of single cells migrating into the matrix and the number of single cells populating each quadrant of the scaffold (Fig. 1G, H). FUS::DDIT3 expression resulted in less growth and infiltration shown by a significantly reduced migration of single cells into the matrix. To determine if eGFP alone affected cellular properties, we also analyzed HT1080 cells expressing eGFP but we observed no effect of eGFP expression compared to WT in HT1080 cells (Additional file 2: Fig. S1C, D). The MLS cell line 1765-92 displayed an overall poor capacity to infiltrate the scaffolds as compared to the different versions of HT1080 cells (Fig. 1I).

### Myxoid liposarcoma scaffolds display a distinct proteomic profile

**Fig. 2:**
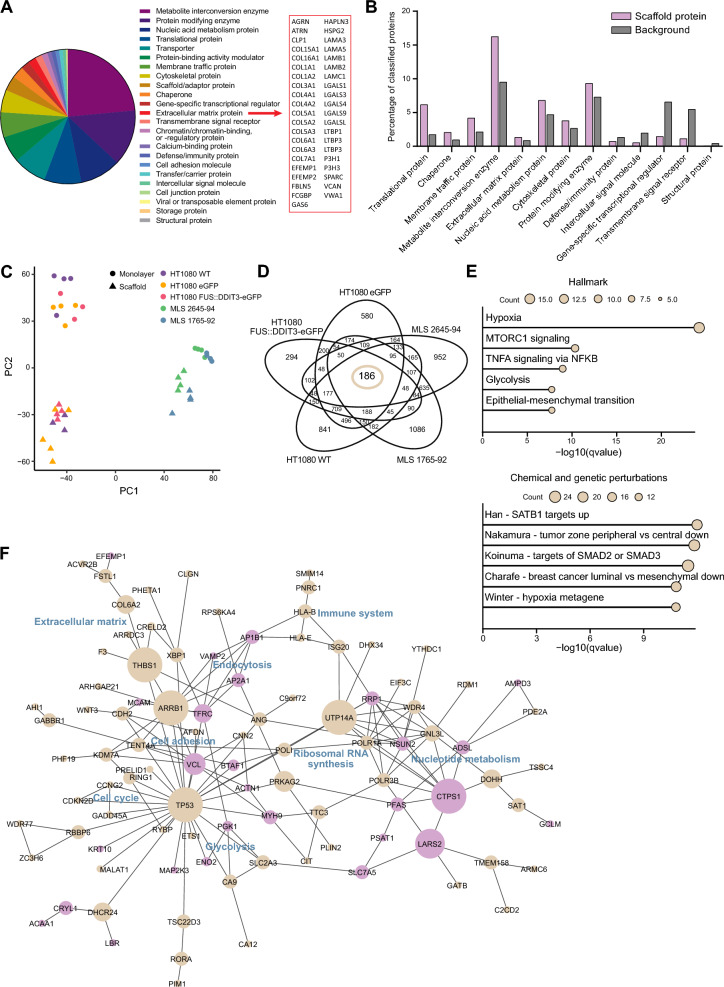
Myxoid liposarcoma scaffold protein composition and scaffold-induced gene expression. **A** Classification of proteins detected in myxoid liposarcoma (MLS) scaffolds (n = 4) using mass spectrometry analysis. Proteins were classified (n = 2172), according to the PANTHER protein classification. All proteins categorized as extracellular matrix protein (n = 41) are listed. **B** Significantly over- and underrepresented categories, comparing MLS scaffold proteins with all protein-coding genes (background) using PANTHER overrepresentation test with a false discovery rate < 0.05. The category order is based on fold enrichment with the highest overrepresentation in MLS scaffolds shown to the left. **C** Principal component analysis of transcriptional profiles based on RNA sequencing of MLS scaffold- and monolayer-cultured cells, respectively, for HT1080 wild-type (WT), HT1080 eGFP, HT1080 FUS::DDIT3-eGFP, MLS 2645-94 and MLS 1765-92, n = 3–5. **D** Venn diagram showing gene regulation overlaps between scaffold- and monolayer-cultured cells in respective cell line. **E** Functional enrichment analysis using the Hallmark and Chemical and genetic perturbations gene set collections for the 186 scaffold-regulated genes. Top 5 categories are shown based on *q*-value. Size of dots indicate gene count. **F** Interaction network of the 186 scaffold-regulated genes generated by Cytoscape based on protein interaction data retrieved from STRING. Node size is based on between-ness centrality, where a large node size indicates many interactions within the network. Purple nodes show proteins expressed in scaffolds. Common properties of adjacent proteins are indicated in blue, assessed from NCBI gene summary and UniProtKB/Swiss-Prot summary for each gene retrieved from GeneCards

To determine the MLS scaffold composition, we analyzed decellularized scaffolds using liquid chromatography-mass spectrometry/mass spectrometry and identified 3090 proteins (Additional file 3: Table S1A). Out of these, 2172 proteins could be functionally categorized, where most proteins were classified as metabolite interconversion enzymes or protein modifying enzymes (Fig. 2A and Additional file 3: Table S1B, C). We identified 41 proteins categorized as extracellular matrix proteins, including 13 collagens, 5 galectins and 5 laminins (Fig. 2A). Eight protein categories were statistically overrepresented comparing MLS scaffold proteins with all protein-coding genes, where translational proteins, chaperones and membrane traffic proteins displayed highest overrepresentation in MLS scaffolds (Fig. 2B and Additional file 3: Table S1D). Five categories were statistically underrepresented among the identified proteins, including gene-specific transcriptional regulators, transmembrane signal receptors and structural proteins.

### Myxoid liposarcoma scaffolds induce a microenvironmental-specific gene expression signature in repopulated tumor cells

To determine general microenvironmental effects on cellular phenotypes, we performed RNA sequencing on cells grown in MLS scaffolds and compared data to monolayer cultures, using HT1080 WT, HT1080 eGFP, HT1080 FUS::DDIT3-eGFP, MLS 1765-92 and MLS 2645-94 cell lines. Unsupervised clustering by principal component analysis showed distinct transcriptional profiles comparing MLS scaffold- with monolayer-cultured cells as well as comparing HT1080 with MLS cell lines (Fig. 2C). We identified a core set of 186 genes that were significantly regulated for all five cell lines in scaffold cultures compared with monolayer cultures (Fig. 2D, detailed in Additional file 4: Table S2A–F for each cell line). Functional enrichment analysis of the 186 regulated genes showed connections to hypoxia, glycolysis, SATB1 regulation and tumor zone peripheral versus central (Fig. 2E and Additional file 4: Table S2G–J). We found a significant overrepresentation (n = 39, Fisher’s exact test, *p* < 0.05) of the 186 scaffold-regulated genes among the 3090 previously identified proteins in the cell-free scaffolds (Additional file 4: Table S2K). To identify central genes and processes among the genes affected by scaffold culture, we generated an interaction network from the 186 MLS scaffold-regulated genes (Fig. 2F). The primary network consisted of 90 genes, where 28 genes (31%) were also expressed as scaffold proteins. Several interacting proteins in the network displayed properties related to cell adhesion, cell cycle, endocytosis, extracellular matrix, glycolysis, immune system, ribosomal RNA synthesis and nucleotide metabolism. Major interaction nodes included *ARRB1*, *CTPS1*, *THBS1*, *TP53* and *UTP14A,* all down-regulated in scaffold-cultured cells compared to monolayer cultures. Of these, *ARRB1* regulates GPCR signaling and affects several pathways, *CTPS1* is involved in nucleotide synthesis and is important for the immune system, *THBS1* is a glycoprotein mediating cell-to-ECM interactions, *TP53* is a known tumor suppressor affecting many processes including the cell cycle and *UTP14A* is involved in ribosomal RNA synthesis. In conclusion, our data show that the cell-free MLS microenvironment induces both intracellular and extracellular processes in growing cancer cell lines.

### FUS::DDIT3 expression modulates cell-to-cell interactions and chromatin remodeling

**Fig. 3:**
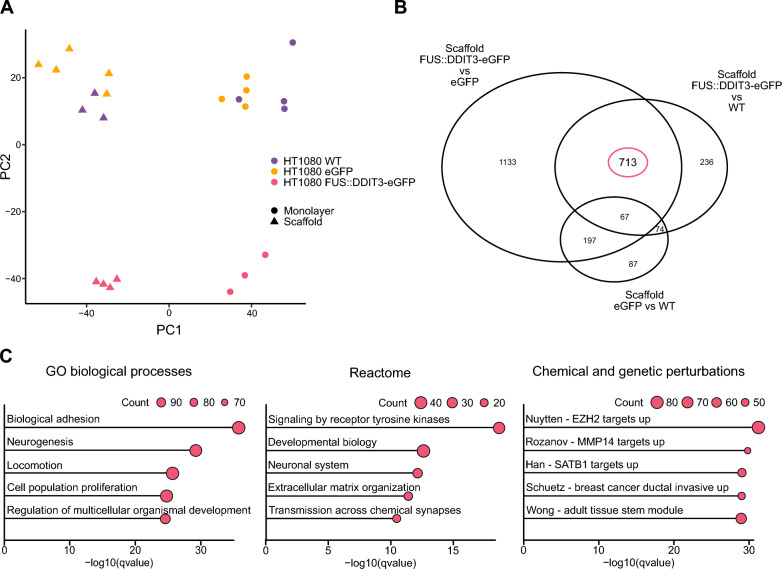
FUS::DDIT3-induced gene expression signatures in myxoid liposarcoma scaffolds. **A** Principal component analysis of transcriptional profiles for HT1080 wild-type (WT), HT1080 eGFP and HT1080 FUS::DDIT3-eGFP cells cultured in either myxoid liposarcoma (MLS) scaffolds or monolayers. n = 3–5. **B** Venn diagram showing gene regulation overlap between HT1080 WT, HT1080 eGFP, and HT1080 FUS::DDIT3-eGFP cells cultured in MLS scaffolds. **C** Functional enrichment analysis using the 713 FUS::DDIT3-regulated genes using GO biological processes, Reactome and Chemical and genetic perturbations gene set collections. Top 5 categories are shown based on *q*-value. Size of dots indicate gene count

To determine specific effects of the *FUS::DDIT3* fusion oncogene in an MLS-specific microenvironment, we compared the transcriptomes of HT1080 cells with and without ectopic FUS::DDIT3-eGFP expression cultured in MLS scaffolds. Unsupervised clustering revealed a distinct separation between HT1080 cells with and without FUS::DDIT3-eGFP expression in addition to the previously identified difference between MLS scaffold- and monolayer-cultured cells (Fig. 3A). Next, we compared HT1080 FUS::DDIT3-eGFP cells with HT1080 WT or HT1080 eGFP cells cultured in scaffolds and identified 713 FUS::DDIT3-regulated genes, where the effect of eGFP expression alone was minor (Fig. 3B and Additional file 5: Table S3A–D). Functional enrichment analysis of the 713 regulated genes identified properties related to cell-to-cell and cell-to-ECM interactions, including biological adhesion, locomotion, proliferation, signaling by receptor tyrosine kinases, extracellular matrix organization, and MMP14 targets (Fig. 3C and Additional file 5: Table S3E–H). In addition, we also detected features related to chromatin remodeling, including EZH2 and SATB1 targets.

### FUS::DDIT3 expression more than microenvironment drives the cellular phenotype in myxoid liposarcoma development

**Fig. 4:**
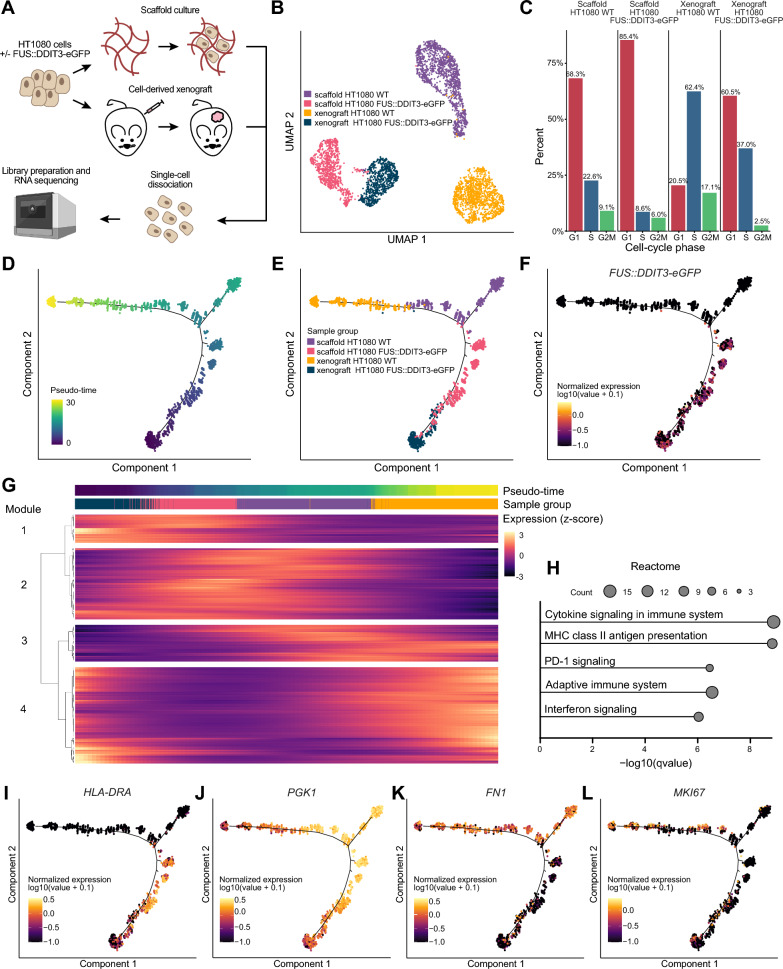
Single-cell analysis of cells grown in myxoid liposarcoma scaffolds and as cell-derived xenografts. **A** Experimental single-cell analysis workflow. **B** Uniform manifold approximation and projection (UMAP) analysis of individual HT1080 cells with and without FUS::DDIT3-eGFP expression grown in myxoid liposarcoma (MLS) scaffolds or as xenografts, n = 1387 (scaffold HT1080 wild-type (WT)), 894 (scaffold HT1080 FUS::DDIT3-eGFP), 1315 (xenograft HT1080 WT), 819 (xenograft HT1080 FUS::DDIT3-eGFP). **C** Bar chart illustrating the percentage of cells in each cell-cycle phase, G1, S and G2/M, based on known cell-cycle-associated genes, for each sample. **D**–**G** Pseudo-time trajectory analysis performed with Monocle 2 using DDR-Tree for dimensional reduction. **D** Distribution of cells along the pseudo-time trajectory is shown. **E** Pseudo-time trajectory with marked sample group. **F** Expression of *FUS::DDIT3-eGFP* across the pseudo-time trajectory (estimated by measuring *eGFP* expression). **G** Significantly differentially expressed genes across pseudo-time are clustered based on co-expression into four modules. The color schemes for pseudo-time and sample group from subplots **D** and **E** are used. **H** Functional enrichment analysis using the Reactome gene set collection for the genes in module 1. Top 5 categories are shown based on q-value. Size of dots indicate gene count. **I**–**L** Expression of selected genes across the pseudo-time trajectory, **I** *HLA-DRA*, **J** *PGK1*, **K** *FN1* and **L** *MKI67*

To further determine the importance of FUS::DDIT3 expression in relation to different microenvironments we performed single-cell gene expression analysis on HT1080 cells with or without FUS::DDIT3 expression grown in MLS scaffolds as well as cell-derived xenografts in mice (Fig. 4A). Dimension reduction analysis showed distinct grouping of single cells based on both microenvironment and FUS::DDIT3 expression, where cells expressing FUS::DDIT3 were more unified in their gene expression pattern compared to cells without FUS::DDIT3 (Fig. 4B). Single-cell analysis also enabled us to assess cell proliferation status, which was decreased by FUS::DDIT3 expression and was also lower in MLS scaffolds compared with xenografts (Fig. 4C). To study cell transitions and define the relationship between different cell types we performed pseudo-time trajectory analysis, where cells are ordered based on their progression through a biological process, such as cell differentiation (Fig. 4D–F and Additional file 2: Fig. S2A,B). Interestingly, FUS::DDIT3 expression unified the cells into overlapping branches regardless of if sarcoma cells were grown in MLS scaffolds or as xenografts in mice (Fig. 4F–G), demonstrating that FUS::DDIT3 expression more than microenvironment determines the transcriptional profile of individual tumor cells. We found 1318 genes that were significantly regulated across the pseudo-time that formed four distinct modules of co-expression based on hierarchical clustering (Fig. 4G and Additional file 6: Table S4A). Next, we performed functional enrichment analysis on each gene module (Fig. 4H and Additional file 6: Table S4B–Q). Module 1 included genes that were highly expressed in the beginning of the pseudo-time, generally in HT1080 cells with FUS::DDIT3 expression cultured in scaffolds or as xenografts. We identified several categories related to immune response and signaling, such as MHC class II antigen presentation, in addition to categories that were already identified in the cell population analysis when comparing HT1080 cells with and without FUS::DDIT3 expression, such as biological adhesion and adipogenesis (Additional file 5: Table S3E–H and Additional file 6: Table S4B–E). In fact, we identified several module 1 genes that are members of the MHC class II, known as human leukocyte antigen (HLA) class II, including *HLA-DRA* (Fig. 4I). Human leukocyte antigen class II genes were also upregulated in FUS::DDIT3-expressing cells in the bulk RNA sequencing data, while HLA class I genes were downregulated (Additional file 5: Table S3A). Additionally, the proto-oncogene *MYC* was found among the module 1 genes (Additional file 2: Fig. S2C). Module 2 genes, with increased expression mainly in scaffold-cultured cells, included glycolytic enzyme PGK1, which was also included among the proteins identified in cell-free scaffolds (Fig. 4J). Among module 3 genes that were highly expressed in HT1080 WT cells was *FN1*, coding for glycoprotein fibronectin, involved in cell adhesion and migration (Fig. 4K). We also identified proliferation marker *MKI67* as a module 4 gene, mostly expressed in xenograft-cultured cells, confirming that cells cultured in scaffolds displayed lower proliferation. *MKI67* also showed higher expression in WT cells compared to FUS::DDIT3-expressing cells (Fig. 4L).

## Discussion

The tumor microenvironment is highly dynamic during tumor development, influencing tumor initiation, progression and metastasis [13]. In addition, ECM composition and architecture affect the behavior of both tumor and stromal cells [36] and has directly been correlated to clinical outcome [37–39]. However, it has been experimentally challenging to study the tumor microenvironment in detail. Cell cultures in monolayers are simple to handle but lack relevant microenvironmental components [40]. In contrast, in vivo mouse models, including PDXs, cell-derived xenografts and genetically engineered mice, can partly mimic the tumor microenvironment. However, these models are often complicated to establish and may require immunocompromised mice. This limits their capability to recapitulate the human microenvironment since the interactions between human immune cells and stromal cells are lost [41, 42]. Three-dimensional in vitro model systems offer new means to recapitulate human tumors [43, 44]. These can be based on inducing aggregation of cells into three-dimensional growth patterns, such as spheroids [44] and organoids [45, 46]. An alternative is to generate three-dimensional scaffolds in which tumor cells can be cultured. Scaffolds can be generated from hydrogels, synthetic materials [44, 47], or human tissues, including tumor tissue [48–52]. Here, we established an experimental in vivo-like scaffold model to study MLS using tumor tissue from PDXs. The choice of using PDX tissue was based on the limited access to patient tumor material as well as the fact that most MLS patients are irradiated prior to surgery, affecting the tumor microenvironment. The establishment of PDX models typically entail numerous tumor passages between immunosuppressed mice combined with histological and molecular verification analysis ensuring that the PDX maintain its original tumor properties [53]. Various decellularization protocols to generate cell-free scaffolds exist [54], where we used a mild detergent wash procedure. The decellularization and repopulation processes were optimized to support cell growth and infiltration and we used 3 weeks to repopulate the MLS scaffolds. Our data and previous studies show that tumor cells need up to 3 weeks to fully infiltrate scaffolds [51, 52]. We cannot rule out that a longer repopulation process is an advantage. For example, some cells may need long time to find their specific microenvironmental niche and then dedifferentiate to MLS-specific subpopulations. However, we observed larger variability between seemingly identical scaffolds when using 7 weeks of growth. Additionally, from an experimental point of view, shorter protocol time is preferred. The scaffold model system has potential application areas beyond the use in this study, including the prediction of patient outcome and to evaluate novel interventions in a patient-specific manner [55–57]. It can also be a resource in attempts to decrease animal use. We speculate that the use of patient-derived scaffolds generated from either tumor tissue or PDXs will be a valuable experimental model system for rare tumor entities, such as the whole family of sarcomas characterized by FET fusion oncogenes [30]. This is particularly interesting when the scaffold can be divided into multiple smaller pieces, as shown in this study. However, the decellularization and repopulation protocol may need to be optimized for different tumor entities.

We identified about 3000 different proteins present in the decellularized MLS scaffolds. As expected, we detected proteins related to ECM, such as collagens and laminins, as well as proteins involved in focal adhesion, including integrins, but also other groups of proteins related to intracellular properties. A limitation with mass spectrometry analysis is that we cannot relate the absolute expression levels between different proteins. Consequently, we cannot distinguish if certain detected collagens are highly abundant as compared to certain intracellular proteins. We cannot rule out that some cell debris remain after decellularization, partly confounding our analysis. However, all proteins used in downstream analysis were reproducibly quantified at a similar level in all decellularized scaffolds generated from different mice with tumors from the same PDX model. Instead, we speculate that the detected intracellular proteins could not be washed away, since they were strongly anchored directly or indirectly to the cell-free microenvironment. Even though the protein class classification is informative, many proteins may display multiple functions and there are likely more proteins that are related to ECM and the microenvironment than the classification system acknowledge. For example, fibronectin (FN1) is categorized as an “intercellular signal molecule” but is also a known extracellular matrix protein and FAK (PTK2) as well as Talin (TLN1) are both involved in focal adhesion but were not categorized in this analysis. Interestingly, in other patient-derived scaffold studies, distinct protein profiles were observed between different tumors of the same entity that could be linked to clinical parameters [51, 58].

Tumor cells distinctly and reproducibly changed their transcriptional profiles when grown in MLS scaffolds compared with monolayers. Growth in scaffolds altered cells expression of genes related to hypoxia, nucleotide metabolism, glycolysis and peripheral versus central tumor zone, all features related to physical barriers and gradients of oxygen and nutrients, typical for in vivo-like model systems [43, 44]. Most other gene expression changes in scaffolds were related to cell-to-cell and cell-to-ECM interactions, including cell adhesion, cell cycle, endocytosis, extracellular matrix, and immune system that are all associated with three-dimensional cell growth. Among the regulated genes in the growing cells, we observed a significant overrepresentation of genes whose translated proteins were also expressed in the scaffold. The underlying reason for this link is unknown, but we speculate that the microenvironment with its unique composition regulates specific gene programs that is, at least partly, directly linked to the gene itself by either positive or negative feedback mechanisms. Our data suggest that cells cultured in MLS scaffolds mimic properties related to in vivo conditions, which is also supported by our single-cell analysis demonstrating that HT1080 cells with FUS::DDIT3 expression generated similar gene signatures regardless of being cultured in MLS scaffolds or as xenografts. The rationale of using HT1080 as a reporter cell line is that these cancer cells tolerate the expression of FUS::DDIT3 and that HT1080 FUS::DDIT3-eGFP cells grow into MLS-like tumors with myxoid ECM production and lipoblast formation when injected into mice [10]. The disadvantages of using HT1080 cells are that they are not of MLS origin and have other driver mutations in genes such as *NRAS* and *IDH1*.

The FUS::DDIT3 fusion oncogene is the major genomic driver event in MLS development. We identified strong connections to epigenetic regulation, including chromatin remodeling, when comparing cells with and without FUS::DDIT3 expression. FET fusion oncoproteins, including FUS::DDIT3, are known to interact with the SWI/SNF and PRC2 chromatin remodeling complexes [30, 59], affecting downstream pathways, such as JAK-STAT signaling [60, 61] and adipocyte differentiation [62]. Interestingly, growth in MLS scaffold also affected processes related to chromatin remodeling in the cells. Additionally, genes involved in adipogenesis, as well as other differentiation processes, were enriched in HT1080 cells expressing FUS::DDIT3 compared to control cells. We also observed downregulation of ECM-related genes *FN1* and *LOXL3* (Additional file 5: Table S3A), genes that are implicated in adipocytic differentiation [63, 64]. These results are in line with the concept that adipogenesis is influenced by interactions with the microenvironment [63]. Collectively, our data supports previous notions that MLS cells have entered initial stages of adipogenesis, but that FUS::DDIT3 blocks terminal adipocytic differentiation [7, 11, 17]. We also detected several gene sets related to cell proliferation and migration associated with FUS::DDIT3-regulated genes. In agreement with these data, we also observed decreased cell infiltration and proliferation in MLS scaffolds for HT1080 cells expressing FUS::DDIT3, where the infiltration capacity of MLS cells was even lower. This agrees with previous data showing reduced proliferation in cells expressing FUS::DDIT3 [26]. The slow-growing nature of MLS tumors can be connected to the cell-free microenvironment, where high abundance of reticular fibers in MLS tumors has been connected to reduced invasiveness [23]. This may explain the low infiltration capacity of MLS cells into scaffolds. Furthermore, the MLS cell lines used are not able to form tumors in mice when subcutaneously injected, indicating that the location of the tumor is critical. Hence, we speculate that MLS is formed at specific locations in the body and that the tumor cells create their own extracellular niche where they gradually adapt to the surrounding tissues. Interestingly, our single-cell clustering data and pseudo-time trajectory analysis collectively showed that FUS::DDIT3 expression differentiated the cells to be more homogenous, strongly suggesting that FUS::DDIT3 drives the cellular MLS phenotype.

Additionally, we observed that both MLS scaffolds and FUS::DDIT3 expression affected immune system-related genes. Our data showed downregulation of HLA class I genes upon FUS::DDIT3 expression and upregulation of HLA class II genes. It has been shown that MLS tissues display lower or no expression of HLA class I genes and proteins [21, 65]. Human leukocyte antigen class I molecules presents peptides to be recognized by CD8+ T cells whereas HLA class II molecules are instead recognized by CD4+ T cells [66], indicating that FUS::DDIT3 affects the T-cell-mediated immune response. Potentially, this effect can also be connected to the adipocytic differentiation process in MLS, as adipocytes express HLA class II genes [67, 68]. A potential problem with these data is that the MLS scaffolds are generated from PDX tumors grown in immunosuppressed mice, where we cannot account for immune system-related biases.

There are some limitations with our study. The scaffolds are generated from the same PDX, providing numerous scaffolds that are seemingly identical to each other. The drawbacks are that we cannot account for intertumoral heterogeneity and the effects of expanding the tumors in immunosuppressed mice are unknown. Another weakness is the use of MLS cell lines. There are few cell lines available, and most are immortalized by SV40 transfection, including the ones applied in this study. It would be interesting to use MLS cells from the PDX model, but we have not succeeded in isolating and growing them ex vivo. Future studies are needed to determine the biological relevance of generating scaffolds from PDX models and the use of different MLS and reporter cells.

## Conclusions

Our experimental approach to use cell-free scaffolds opens new possibilities to study the properties of the MLS microenvironment and how tumor cells interact with each other as well as with the ECM. The scaffold platform is suitable for drug testing since numerous scaffolds can be prepared from the same tumor. Our work in the context of MLS development has uncovered that the cell-free microenvironment and FUS::DDIT3 expression both activates gene programs related to cell-to-cell and cell-to-ECM interactions, as well as chromatin remodeling, immune response, and metabolism. Data also indicate that the FUS::DDIT3 expression more than the microenvironment affects differentiation towards an early adipocytic phenotype, where MLS cells can create their own extracellular niche.

### Supplementary Information

**Additional file 1. **Supplementary materials and methods.

**Additional file 2:** **Figure S1.** Characterization and cell growth using myxoid liposarcoma scaffolds as an in vivo-like model system. **Figure S2.** Pseudo-time trajectory analysis.

**Additional file 3: Table S1.** Protein characterization of cell-free myxoid liposarcoma scaffolds.

**Additional file 4: Table S2.** Regulated genes between scaffold-cultured and monolayer-cultured cells.

**Additional file 5: Table S3.** Regulated genes between scaffold-cultured HT1080 FUS::DDIT3-eGFP cells and control cell lines.

**Additional file 6: Table S4.** Genes differentially expressed across pseudo-time.

## Data Availability

Gene expression data are available through Gene expression omnibus (GEO) for bulk RNA sequencing (GSE230773, https://www.ncbi.nlm.nih.gov/geo/query/acc.cgi?acc=GSE230773) and for the 10 × Genomics single-cell data (GSE191132, https://www.ncbi.nlm.nih.gov/geo/query/acc.cgi?acc=GSE191132). The mass spectrometry proteomics data have been deposited to the ProteomeXchange Consortium via the PRIDE [69] partner repository, https://www.ebi.ac.uk/pride/, with the dataset identifier PXD023792.

## Abbreviations

ECM: Extracellular matrix
FET: FUS, EWSR1, TAF15
HLA: Human leukocyte antigen
MLS: Myxoid liposarcoma
PDX: Patient-derived xenograft
WT: Wild-type
