# Supplementary material for: Deciphering the role of FUS::DDIT3 expression and tumor microenvironment in myxoid liposarcoma development
Source: J Transl Med. 2024 Apr 26;22:389. doi: 10.1186/s12967-024-05211-w (PMC11046918; doi:10.1186/s12967-024-05211-w)
Supplement: Supplementary file 1 — Additional file 1. Supplementary materials and methods. [file 12967_2024_5211_MOESM1_ESM.docx]

# Supplementary materials and methods

## RNA sequencing

Total RNA was extracted from cell cultures in monolayers using either RNeasy Micro Kit or miRNeasy Micro kit, according to manufacturer’s instructions using DNase treatment (all from Qiagen, Hilden, Germany). Cells cultured in MLS scaffolds were washed twice with distilled PBS solution (Thermo Fisher Scientific, Waltham, MA, USA) and lysed directly in RLT lysis buffer (Qiagen). Samples were homogenized using stainless steel beads in TissueLyzer II (both Qiagen) for 2x5 min at 25 Hz. Samples were visually checked and if not shattered, homogenization was repeated for another 5 min until completely lysed. Samples were centrifuged at 4°C for 1 min at 10,000 rpm and the lysates were then transferred to clean microcentrifuge tubes. RNA extraction was then performed using RNeasy Micro Kit including DNase treatment, according to manufacturer’s instructions. RNA concentration was quantified with either NanoDrop (Thermo Fisher Scientific) or Qubit 3.0 Fluorometer using Qubit RNA HS Assay Kit (Thermo Fisher Scientific). RNA integrity was assessed with either RNA 6000 Nano Kit on a 2100 Bioanalyzer or DNF-472 kit on a Fragment Analyzer (all Agilent Technologies, Santa Clara, CA, USA).

RNA sequencing was performed according to the Smart-Seq2 protocol [1] with some modifications [2] where 10 ng total RNA was used in the reverse transcription step and preamplification was performed for 15-18 cycles. Concentration and quality of preamplified cDNA and final libraries were assessed with Qubit dsDNA High Sensitivity Assay (Thermo Fisher Scientific) and DNF-474 HS NGS Fragment kit on a Fragment Analyzer or High Sensitivity DNA Kit on a 2100 Bioanalyzer Instrument (all Agilent). For each sequencing run, libraries were pooled equimolarly and sequencing was performed paired-end on either a MiniSeq instrument using MiniSeq High Output Reagent kit or a NextSeq500 instrument using NextSeq 500 Mid Output v2 kit (all Illumina, San Diego, CA, USA), according to the manufacturer’s instructions. The NextSeq analysis was performed at Clinical Genomics Gothenburg, Science for Life Laboratory, Sahlgrenska Academy, University of Gothenburg (Gothenburg, Sweden).

Read alignment was performed using STAR RNA-seq aligner v2.6 [3] with ENSEMBL GRCh38 assembly as the reference genome. Read count matrices were generated using the HTSeq python framework v0.9.1 [4]. Genes with a total count number less than 10 were excluded from downstream analyses. Differential expression was analyzed using the R package DESeq2, based on shrink estimation for dispersion and fold-change using a negative binomial distribution model [5]. Adjusted *p*-values were calculated using the Benjamini-Hochberg method and genes at least two-fold regulated (adjusted p-value ≤ 0.05) were analyzed in downstream analysis. Functional enrichment analysis was performed by comparing gene lists to gene set collections retrieved from the molecular signature database (MSigDB) v7.4 [6, 7] using the enricher function of R package clusterProfiler v3.14.3 [8]. The enrichment analysis was modified to match the methodology used by the MSigDB web tool by redefining the background as the totality of human genes curated by the HGNC (HUGO Gene Nomenclature Committee). Network analysis was performed using Cytoscape v3.8.0 [9] with interaction data retrieved from the STRING database [10] using the StringApp v1.5.1 [11], implemented in R using the RCy3 package. Central proteins within the network were defined by betweenness centrality calculated with the Network Analyzer tool [12]. Shared properties of genes were assessed from NCBI gene summary and UniProtKB/Swiss-Prot summary accessed from GeneCards [13]. Identified scaffold proteins were overlapped with scaffold-regulated genes and the overlapping probability was evaluated with a Fisher’s exact test using all protein-coding genes as background.

## Single-cell RNA sequencing

For MLS scaffolds, single cells were detached from the scaffold using 0.25% trypsin (Thermo Fisher Scientific). For xenografts, tumors were minced on 60 mm plastic dishes (Thermo Fisher Scientific) and dissociated by incubation in 5 mL media, containing 1x collagenase/hyaluronidase (STEMCELL Technologies, Vancouver, Canada) for 4 h at 37°C and 100 rpm using a Incu-Shaker 10L. The solution was passed through a 19G needle (Becton Dickinson, Franklin Lakes, NJ, USA) and filtered through a 40 µm cell strainer (Corning Life Sciences, Amsterdam, The Netherlands). Cell suspension was centrifuged for 5 min at 300 g and then resuspended in 10 mL distilled PBS supplemented with 10 % fetal bovine serum (Thermo Fisher Scientific). Viable cells were enriched with Dead Cell Removal Kit (Miltenyi Biotec, Bergisch Gladbach, Germany), according to the manufacturer’s instructions. Cells were counted with a Moxi Z automated cell counter (Orflo, Ketchum, ID, USA).

After cell preparation, single cell suspensions were processed immediately and incorporated into Single Cell 3’ gel beads on a Chromium instrument (10x Genomics, Pleasanton, CA, USA). Sequencing libraries were prepared according to the manufacturer’s instructions using the version 2 chemistry (10x Genomics). The quality of cDNA and sequencing libraries was assessed using the DNF-474 HS NGS Fragment kit on a Fragment Analyzer. Sequencing was performed on Nextseq500 instrument using NextSeq 500 MidOutput kit (all Illumina), according to the manufacturer’s instructions at Clinical Genomics Gothenburg, Science for Life Laboratory, Sahlgrenska Academy, University of Gothenburg. Libraries were analyzed using paired-end sequencing with single indexes, according to the Single Cell 3’ v2 kit instructions.

Single-cell data analysis was performed using Cell Ranger v4.1.1 (10x Genomics) and the resulting barcode matrices were analyzed in R using the Seurat package v4.0.3 [14]. Cells with less than 2000 or more than 8000 expressed genes as well as genes expressed in less than 1% of all cells were excluded from downstream analysis. To eliminate necrotic and apoptotic cells, cells with a mitochondrial mRNA to total mRNA ratio larger than 10% were removed. Data normalization and scaling were performed using the Seurat functions *NormalizeData* and *ScaleData* with the default parameters. Data variability linked to the percentage of mitochondrial RNA was regressed in this step. Unsupervised clustering of the cells was performed on the first 15 principal components using the nearest-neighbor algorithm followed by dimensionality reduction using uniform manifold approximation and projection algorithm. Cell grouping was performed using the Leiden community detection algorithm implemented in the Seurat function *FindClusters* with a resolution of 1.2. Differential gene expression analysis was performed using the R package MAST v1.18.0 [15] implemented in the Seurat function *FindMarkers* with the number of expressed genes as a latent variable for genes that were expressed in at least 2.5% of the cells in any group. *p*-values were adjusted for multiple testing using Bonferroni correction and considered statistically significant if the adjusted *p*-value < 0.05.

Pseudo-time ordering and developmental trajectories were performed with Monocle 2 DDR-Tree v2.20.0 [16], Slingshot v2.0.0 [17] and SCORPIUS v1.0.8 [18]. The Monocle pseudo-time ordering was generated using the top 1000 differentially expressed genes between single cell clusters determined with the density peak algorithm using the threshold settings rho = 1 and delta = 20. Differential gene expression across the pseudo-time was tested using generalized linear models for the 1500 most differentially expressed genes, based on *q*-value between clusters as determined by density peak clustering implemented in Monocle. Genes that were significantly regulated across pseudo-time (*q*-value < 0.1) were visualized in a heatmap and grouped into modules of co-expression using hierarchical clustering. Functional enrichment analysis was performed as described for the bulk RNA sequencing data.

# References

1. Picelli S, Faridani OR, Bjorklund AK, Winberg G, Sagasser S, Sandberg R: **Full-length RNA-seq from single cells using Smart-seq2.** *Nat Protoc* 2014, **9:**171-181.

2. Lindén M, Thomsen C, Grundevik P, Jonasson E, Andersson D, Runnberg R, Dolatabadi S, Vannas C, Luna Santamaría M, Fagman H, et al: **FET family fusion oncoproteins target the SWI/SNF chromatin remodeling complex.** *EMBO Rep* 2019, **20:**e45766.

3. Dobin A, Davis CA, Schlesinger F, Drenkow J, Zaleski C, Jha S, Batut P, Chaisson M, Gingeras TR: **STAR: ultrafast universal RNA-seq aligner.** *Bioinformatics* 2012, **29:**15-21.

4. Anders S, Pyl PT, Huber W: **HTSeq--a Python framework to work with high-throughput sequencing data.** *Bioinformatics* 2015, **31:**166-169.

5. Love MI, Huber W, Anders S: **Moderated estimation of fold change and dispersion for RNA-seq data with DESeq2.** *Genome Biol* 2014, **15:**550.

6. Subramanian A, Tamayo P, Mootha VK, Mukherjee S, Ebert BL, Gillette MA, Paulovich A, Pomeroy SL, Golub TR, Lander ES, Mesirov JP: **Gene set enrichment analysis: a knowledge-based approach for interpreting genome-wide expression profiles.** *Proc Natl Acad Sci U S A* 2005, **102:**15545-15550.

7. Liberzon A, Subramanian A, Pinchback R, Thorvaldsdóttir H, Tamayo P, Mesirov JP: **Molecular signatures database (MSigDB) 3.0.** *Bioinformatics* 2011, **27:**1739-1740.

8. Yu G, Wang LG, Han Y, He QY: **clusterProfiler: an R package for comparing biological themes among gene clusters.** *OMICS* 2012, **16:**284-287.

9. Shannon P, Markiel A, Ozier O, Baliga NS, Wang JT, Ramage D, Amin N, Schwikowski B, Ideker T: **Cytoscape: a software environment for integrated models of biomolecular interaction networks.** *Genome Res* 2003, **13:**2498-2504.

10. Szklarczyk D, Gable AL, Lyon D, Junge A, Wyder S, Huerta-Cepas J, Simonovic M, Doncheva NT, Morris JH, Bork P, et al: **STRING v11: protein-protein association networks with increased coverage, supporting functional discovery in genome-wide experimental datasets.** *Nucleic Acids Res* 2019, **47:**D607-d613.

11. Doncheva NT, Morris JH, Gorodkin J, Jensen LJ: **Cytoscape StringApp: Network Analysis and Visualization of Proteomics Data.** *J Proteome Res* 2019, **18:**623-632.

12. Assenov Y, Ramirez F, Schelhorn SE, Lengauer T, Albrecht M: **Computing topological parameters of biological networks.** *Bioinformatics* 2008, **24:**282-284.

13. Stelzer G, Rosen N, Plaschkes I, Zimmerman S, Twik M, Fishilevich S, Stein TI, Nudel R, Lieder I, Mazor Y, et al: **The GeneCards Suite: From Gene Data Mining to Disease Genome Sequence Analyses.** *Current Protocols in Bioinformatics* 2016, **54:**1.30.31-31.30.33.

14. Hao Y, Hao S, Andersen-Nissen E, Mauck WM, 3rd, Zheng S, Butler A, Lee MJ, Wilk AJ, Darby C, Zager M, et al: **Integrated analysis of multimodal single-cell data.** *Cell* 2021, **184:**3573-3587.e3529.

15. Finak G, McDavid A, Yajima M, Deng J, Gersuk V, Shalek AK, Slichter CK, Miller HW, McElrath MJ, Prlic M, et al: **MAST: a flexible statistical framework for assessing transcriptional changes and characterizing heterogeneity in single-cell RNA sequencing data.** *Genome Biol* 2015, **16:**278.

16. Qiu X, Mao Q, Tang Y, Wang L, Chawla R, Pliner HA, Trapnell C: **Reversed graph embedding resolves complex single-cell trajectories.** *Nat Methods* 2017, **14:**979-982.

17. Street K, Risso D, Fletcher RB, Das D, Ngai J, Yosef N, Purdom E, Dudoit S: **Slingshot: cell lineage and pseudotime inference for single-cell transcriptomics.** *BMC Genomics* 2018, **19:**477.

18. Cannoodt R, Saelens W, Sichien D, Tavernier S, Janssens S, Guilliams M, Lambrecht B, Preter KD, Saeys Y: **SCORPIUS improves trajectory inference and identifies novel modules in dendritic cell development.** bioRxiv; 2016.
