# Supplementary material for: Deciphering the role of FUS::DDIT3 expression and tumor microenvironment in myxoid liposarcoma development
Source: J Transl Med. 2024 Apr 26;22:389. doi: 10.1186/s12967-024-05211-w (PMC11046918; doi:10.1186/s12967-024-05211-w)
Supplement: Supplementary file 2 — Additional file 2: Figure S1. Characterization and cell growth using myxoid liposarcoma scaffolds as an in vivo-like model system. Figure S2. Pseudo-time trajectory analysis. [file 12967_2024_5211_MOESM2_ESM.docx]

# Supplementary Figures


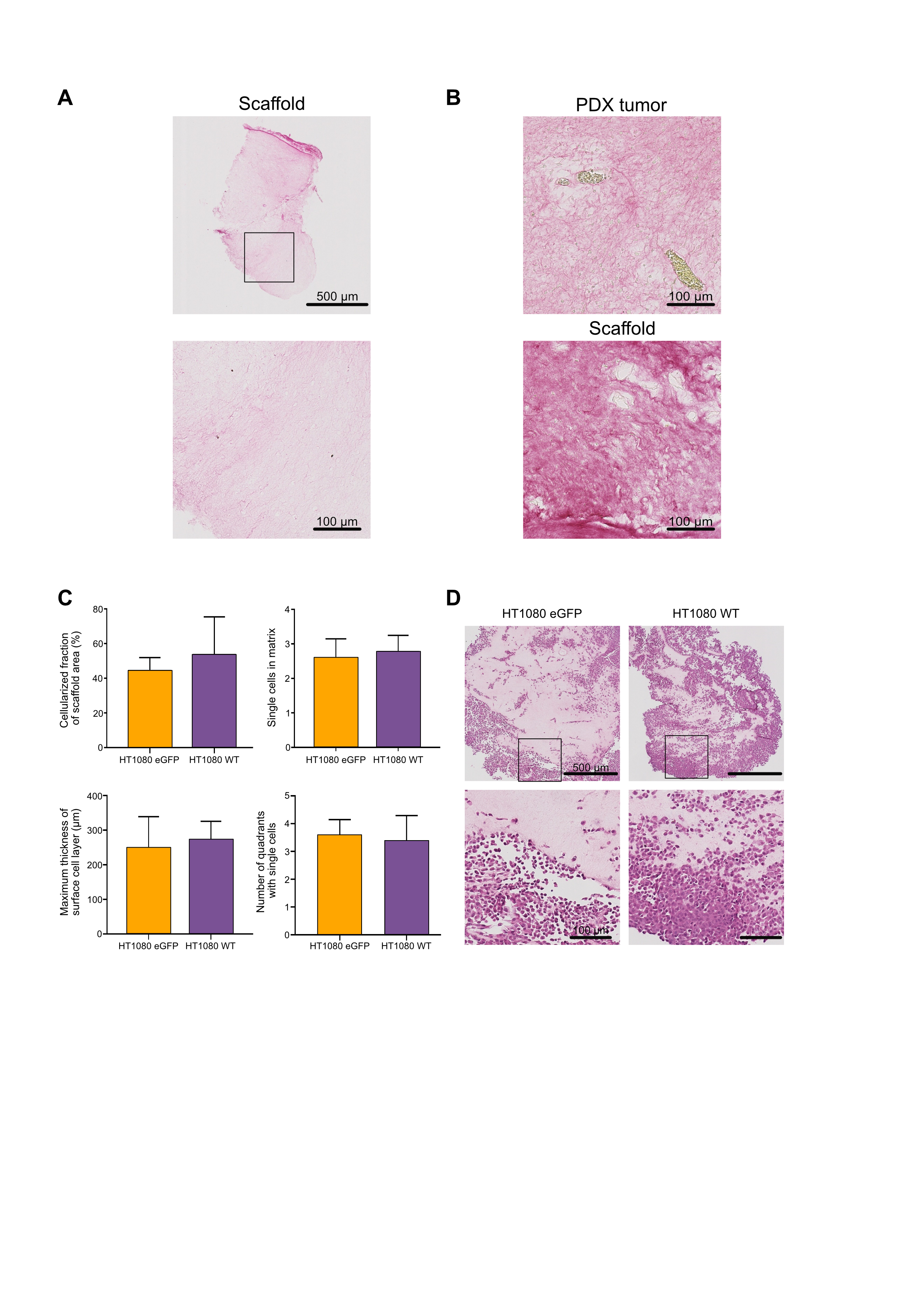


**Figure S1.** **Characterization and cell growth using myxoid liposarcoma scaffolds as an *in vivo*-like model system.** A) Hematoxylin and eosin staining of decellularized MLS scaffold. Image below is a representative magnification. No nuclei were observed after two rounds of washing. B) Picro-Sirius Red staining showing collagen structure of myxoid liposarcoma (MLS) patient-derived xenograft (PDX) tumor and decellularized MLS scaffold. Collagen is shown in red. C) Comparison of HT1080 cells with and without ectopic eGFP expression cultured in scaffolds for 3 weeks and quantification of cellularized fraction of the scaffold area, maximum thickness of surface cell layer, single cells migrating into the matrix and number of single cells populating each quadrant. Mean ± SEM is shown, ­n = 5, Student’s t-test. No differences were statistically significant *p* < 0.05. D) Hematoxylin and eosin staining of scaffolds repopulated with HT1080 eGFP and HT1080 wild-type (WT) cell lines both cultured for 3 weeks. Images below are representative magnifications.


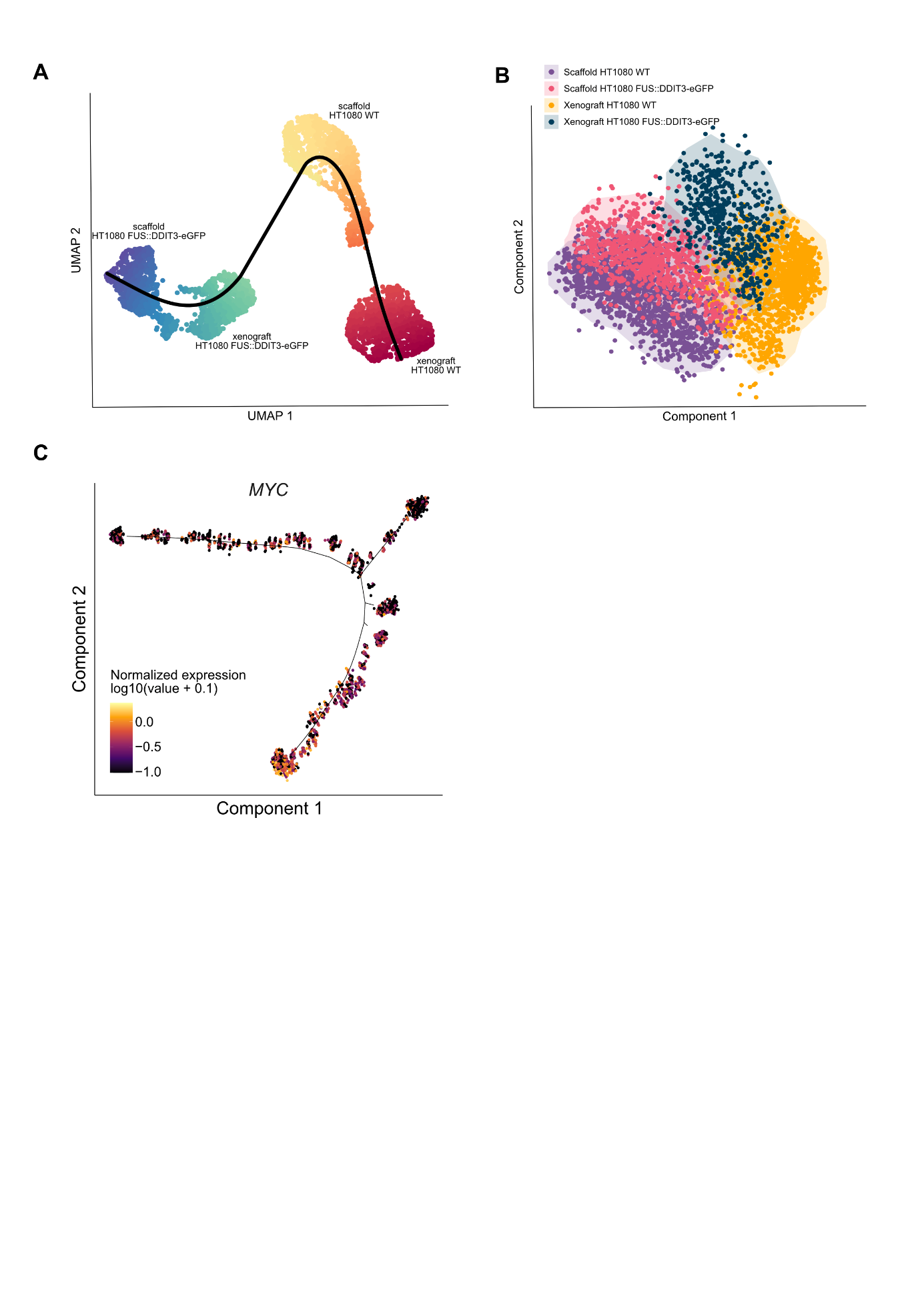


**Figure S2. Pseudo-time trajectory analysis.** A) Pseudo-time trajectory analysis using the slingshot algorithm. Visualized by UMAP analysis. B) Pseudo-time trajectory analysis using the SCORPIUS algorithm. Visualized by landmark multi-dimensional scaling. C) Expression of *MYC* across the pseudo-time trajectory resulting from analysis using the Monocle 2 algorithm.
